# Lyme and Dopaminergic Function: Hypothesizing Reduced Reward Deficiency Symptomatology by Regulating Dopamine Transmission

**DOI:** 10.15761/JSIN.1000163

**Published:** 2017-05-11

**Authors:** Kenneth Blum, Edward J Modestino, Marcelo Febo, Bruce Steinberg, Thomas McLaughlin, Lyle Fried, David Baron, David Siwicki, Rajendra D Badgaiyan

**Affiliations:** 1 Department of Psychiatry, McKnight Brain Institute, University of Florida School of Medicine, Gainesville, FL., USA; 2 Division of Neuroscience Research & Addiction Therapy, Shores Treatment & Recovery Center, Port Saint Lucie, FL., USA; 3 Department of Clinical Psychology and Addiction, Institute of Psychology, Eötvös Loránd University, Budapest, Hungary; 4 Division of Addiction Services, Dominion Diagnostics, LLC., North Kingstown, RI, USA; 5 Department of Psychiatry and Behavioral Sciences, Keck School of Medicine, University of Southern California, Los Angeles, CA., USA; 6 Department of Psychiatry, Wright State University, Dayton, Oh., USA; 7 Department of Precision Medicine, Geneus Health LLC, San Antonia, TX, USA; 8 Department of Psychology, Curry College, Milton, MA, USA; 9 Center for Psychiatric Medicine North Andover, MA, USA

**Keywords:** lyme disease, Borrelia burgdorferi, dopamine, reward deficiency syndrome

## Abstract

The principal vector of Lyme disease in the United States is Ixodes scapularis: black legged or deer ticks. There is increased evidence that those infected may be plagued by anxiety or depression as well. Researchers have identified transcripts coding for two putative cytosolic sulfotransferases in these ticks, which recognized phenolic monoamines as their substrates. It is hypothesized that protracted Lyme disease sequelae may be due to impairment of dopaminergic function of the brain reward circuitry. The subsequent recombinant proteins exhibited sulfotransferase function against two neurotransmitters: dopamine and octopamine. This, in itself, can reduce dopamine function leading to many Reward Deficiency Syndrome behaviors, including depression and possibly, anxiety. In fact, it was shown that activity of Ixosc Sult 1 and Sult 2 in the Ixodid tick salivary glands might contain inactivation of the salivation signal through sulfonation of either dopamine or octopamine. This infraction results in a number of clinically observed mood changes, such as anxiety and depression. In fact, there are common symptoms observed for both Parkinson and Lyme diseases. The importance of understanding the mechanistic and neurobiological effects of Lyme on the central nervous system (CNS) provides the basis for pro-dopamine regulation as a treatment. WC 195

## Hypothesis

It is hypothesized that protracted Lyme disease sequelae may be due to impairment of dopaminergic function of the brain reward circuitry. This infraction results in a number of clinically observed mood changes, such as anxiety and depression. The importance of understanding the mechanistic and neurobiological effects of Lyme on the central nervous system (CNS) provides the basis for pro-dopamine regulation as a treatment.

## Introduction

Lyme disease (Lyme borreliosis) is an infectious disease caused by Borrelia bacteria. The principal vector of Lyme disease in the United States is Ixodes scapularis: black legged or deer ticks [1]. The typical symptom is a developing area of redness (erythema migrans), which starts at the site of a tick bite approximately seven days after the initial bite. The rash is neither itchy nor painful. An estimated 25–50% of those infected never develop a rash. Other symptoms may include the following: fever, headache, and lethargy. If left untreated, symptoms may worsen such as the loss of face mobility, joint pain, severe headaches accompanied by neck stiffness, or heart palpitations, amongst others. Symptoms can continue several months to even years later, where continued episodes of joint pain and swelling may occur. Occasionally, individuals may develop shooting pains or tingling in their arms and legs (Restless Leg Syndrome). Even with appropriate treatment protocols, approximately 10–20% of those infected may develop joint pain, memory issues, and increased lethargy for a minimum of six months. There is increased evidence that those infected may be plagued by Neuropsychiatric Disorders (anxiety, depression etc.).

It is well known that Lyme Disease (Lyme borreliosis), progresses from an initial skin infection to a disabling multi-systemic illness. It is of interest that today the most common vector-borne infection in the United States, Lyme disease is increasing in incidence and geographic spread [1]. Lyme disease has dermatologic, arthritic, ophthalmologic, cardiac, neurologic, and psychiatric manifestations [2]. There are common features between Lyme disease and Syphilis. Specifically, it’s protean manifestations, in its spirochetal etiology, and in its course (early skin localization and rapid invasion of the central nervous system [CNS]), Lyme disease is similar to syphilis [3]. Similar to syphilis, early recognition is important to prevent an acute, treatable illness from becoming a chronic or relapsing one. One current issue is that diagnostic tests are not reliable, and as such physicians must rely on clinical presentation as the basis for diagnosis. Since many of the symptoms of Lyme disease involve the CNS, patients with Lyme disease may be referred to psychiatrists both before and after diagnosis.

The first report in the United Sates of a tick-induced erythema migrans rash was in 1970 [4]. In 1977 “Lyme arthritis” was reported by Steere *et al.* [5]; Furthermore, Steere and associates based their report on an epidemiological investigation of an outbreak of juvenile rheumatoid arthritis in Connecticut. In 1978 the link between Lyme arthritis and the bite of an Ixodes tick [6,7] was recognized. Importantly, Burgdorfer *et al.* isolated the etiologic agent of Lyme disease from an Ixodes tick--a spirochete known as B. burgdorferi [4]. Early in the history of Lyme disease, aspirin and nonsteroidal anti-inflammatory agents were used for symptoms that emerged after the erythema migrans rash [8]. Over a number of years penicillin was shown to shorten the duration of illness, thus supporting an infectious etiology. It has been reported that while short courses (10 days) of oral or intravenous antibiotics were recommended at first, currently it is recognized that some patients benefit from longer courses (6 weeks or longer) or repeated treatments [9–11].

## Centers for Disease Control (CDC) Criteria for Diagnosis

The CDC began to review Lyme Disease in 1982, and in 1991 Lyme disease became nationally reportable [12,13] which involves: 1) a physician-diagnosed erythema migrans rash of at least 5 cm in diameter or 2) laboratory confirmation of exposure to *B. burgdorferi* and at least one systemic manifestation. In addition, systemic manifestations must be either musculoskeletal (arthritis), neurologic (lymphocytic meningitis, cranial neuritis, radiculopathy, encephalomyelitis with intrathecal antibody production), or cardiac (second- or third-degree atrioventricular conduction delays). Moreover, laboratory confirmation requires the isolation of *B. burgdorferi*, the demonstration of diagnostic levels of *B. burgdorferi* immunoglobulin (1g) M or IgG antibodies in serum or CSF, or a rising specific antibody titer on serum samples obtained from acutely ill and convalescent patients. It is noteworthy, that about one-third of patients do not recall the erythema migrans rash and serologic testing may be unreliable [14].

## Neuropsychiatric disorders induced by lyme

Over the years there have been many case reports, that have linked a variety of neurologic syndromes to late Lyme disease; these include blindness [15], progressive dementias [16], seizure disorders [17], the extrapyramidal disorders [18], and other neurological syndromes and disorders [19–21]. There are many reports indicating that Lyme patients present with irritability, mood lability, or depression [22–27]. German scientists revealed that “psychiatric manifestations can at times be predominant, ranging from agitated depressive states to the clinical picture of dementia [28].” Along these lines, Kohler suggested that in some Lyme patients there is a “staging of psychiatric symptoms”, with depression occurring in early CNS disease and organic mood and psychotic disorders occurring in late-stage disease [29]. It is accepted that up to 40% of patients with Lyme disease develop neurologic involvement of either the peripheral or central nervous system. It is of interest that a broad range of psychiatric reactions have been associated with Lyme disease, including paranoia, dementia, schizophrenia, bipolar disorder, panic attacks, major depression, anhedonia, anorexia nervosa, and obsessive-compulsive disorder.

With this brief background we are compelled to suggest that these broad psychiatric reactions may involve a “hypodopaminergia”.

## The dopamine connection

It is of interest that a group of scientists has been investigating methods to screen for tick dopamine receptors as a way to control for vector spread of Lyme disease [30]. Current improvements in transcriptome research have discovered many distinctive proteins expressed in the salivary glands of hard ticks, in which the bulk have no known function, and contain several novel protein families. Recently, Pichi *et al.* [31] identified transcripts coding for two putative cytosolic sulfotransferases in these ticks, which recognized phenolic monoamines as their substrates. They characterized the genetic expression of these two cytosolic sulfotransferases during the tick life progression and also the enzymatic properties of similar recombinant proteins. Remarkably, the subsequent recombinant proteins exhibited sulfotransferase function against two neurotransmitters: dopamine and octopamine. This, in itself, can reduce dopamine function leading to many RDS behaviors, including depression and possibly, anxiety. In fact, it was shown that activity of Ixosc Sult 1 and Sult 2 in the Ixodid tick salivary glands might contain inactivation of the salivation signal through sulfonation of either dopamine or octopamine [32].

In consideration of past years, innovative developments have been described in the role of Toll-like receptors (TLRs) in the chronic inflammation as seen in rheumatic diseases. In particular, the inhibitory activity of TLR10 has been established. Receptors that improve the utility of TLRs, as well as numerous TLR inhibitors, have been recognized. Additionally, the primary role of the microbiome and TLRs in the onset of rheumatic diseases has also been reported. Joosten *et al*. [33] reviewed new insights on the role of TLRs in many inflammatory joint diseases (i.e., rheumatoid arthritis, systemic lupus erythematosus, gout, Lyme arthritis) focusing on the signal mechanisms facilitated by the Toll-IL-1 receptor (TIR) domain, the exogenous and endogenous TLR ligands, and the present and potential therapeutic approaches to target TLR signaling in rheumatic diseases.

## Possible induction of RDS in lyme

There is evidence that Lyme can affect the function of TLRs, which are natural immunity-related receptors. Several research studies have specified the participation of TLRs in neurophysiology and neuropathology. One particular study indicated that TLR3 controls hippocampal memory, and is exceedingly expressed in the mesolimbic dopamine system, implying that TLR3 signaling may control alcohol ingestion. This study assessed the potential role of TLR3 in alcohol intake pattern. Jang *et al.* [34] utilized adult BalbC wild-type mice and TLR3 knockout mice testing two-bottle alcohol preference over a period of 15 days and one-bottle 2- or 4-hour drinking in the dark over a period of 4 days. The 10% alcohol consumption rate of TLR3 knockout mice amplified on the 24-hour free-choice test. Our results maintain a possible monitoring role of TLR3 in alcohol consumption. There is also evidence of opiate abuse and the expression of Toll-like Receptor 9, especially in HIV-1 infected patients [35]. Opiate abuse and HIV-1 have been designated as interrelated epidemics, and though the introduction of combined anti-retroviral therapy, further opiate abuse happens to affect greater neurologic and cognitive deficits. The central nervous system (CNS) is especially susceptible to interactive opiate/HIV-1 outcomes, in part, because of the distinctive reactions of microglia and astroglia. Neurons are responsible for both behavior and cognition. However, HIV-1 infection and replication in the brain principally affects microglia while astroglia and glial progenitors are latently infected. Therefore, neuronal dysfunction results from cellular and viral toxins from such HIV-1 infected/exposed glia. Notably, subgroups of glial cells, such as oligodendrocytes and neurons express μ-opioid receptors and can, therefore, become direct targets for heroin and morphine (the major metabolite of heroin in the central nervous system) – agents that preferentially activate μ-opioid receptors. Opiates also enhance synaptodendritic damage and they decrease synaptic connectivity, thus impacting cognitive deficits and RDS patterns. Opioid signaling and interactions with HIV-1 are contextual, differing widely among cell types and even within subtypes. For instance, astroglia in a single brain region are heterogeneous in their expression of μ-, δ-, and κ-opioid receptors, in addition to CXCR4 and CCR5, and Toll-like receptors. Understanding how opiate use exacerbates RDS and therefore requires distinct definition of targets engaged by opiates in each cell type, and among brain regions [36].

The message herein is that Lyme, through its dangerous vectors, can induce neurological impairment, especially affecting the neurochemical integrity of the brain reward circuitry. It is our position that Lyme induction antagonizes the function of dopamine by inactivation and enhancement of pro-inflammatory cytokines, bringing about a deficiency of Toll–Receptors (*i.e.,* TLR3). This will have profound effects and can induce unwanted anxiety and depression.

Since it is now known that a number of dopaminergic-related gene polymorphisms are linked to depressive symptoms, it is tantamount to at least consider the possibility of utilizing a complex known to balance dopaminergic activity in the CNS. Although there is no current research showing the possibility of reduction of anxiety or depression induced by Lyme, the rationale seems sound.

In this regard, Willuhn *et al.* [37] found that as dopaminergic function decreases, cocaine consumption and other addictive behaviors increase, including the behaviors that are irrelevant of substance abuse. Long-term cocaine abuse is linked to D2 and D3 receptor decrease and lowered stimulation of the occipital cortex and cerebellum. In particular, dopamine agonist therapy, which conserves and repairs dopamine functioning, may potentially serve as a successful approach to relapse prevention in psychoactive drug and behavioral addictions. After a lengthy review of Medication Assisted Treatment (MAT), we have pinpointed the failures of glutaminergic medications, specifically in the chronic treatment of Reward Deficiency Syndrome (RDS) behaviors. Both neurogenetics and epigenetics are incredibly important in addiction treatment response and clinical outcomes. According to scientific research, we suggest the use of the term “dopamine agonist therapy” for long-term and concur the short-term use of “dopamine antagonistic therapy” with warning.

The literature is split between robust examples of genetic and epigenetic links to relapse and the apparent prevention thereof. Certainly, even if the resultant RDS behaviors (*i.e.,* anxiety and depression) could lead to subsequent substance and non-substance addiction seeking as induced by Lyme, mimicking either genetic or epigenetic insults, our proposal of utilizing KB220z seems parsimonious.

In fact, our proposal is extended to the fact that the hospitalization and perhaps mortality due to enhanced relapse of RDS seeking should consider prevention tactics such as the Neuroadaptagen–Amino-Acid therapeutic (KB220), now referred to as the Natural Assisted Treatment™ [38].

## Conclusion

The reasoning behind our proposal connects to the understanding of neuro–mechanisms, relating “dopamine homeostasis” to addiction recovery – drug and non–drug addictive behaviors. Fortunately, the addicted brain, specifically DRD2 A1 carriers, favors Neuroadaptagen-Amino-Acid therapy [38] due to an increased sensitivity to promote dopaminergic activity. This occurs as a result of dopamine synthesis: DRD2 A1 allele carriers display augmented striatal activity of L-amino acid decarboxylase. Ultimately, future research should be focused on the query of the role of “dopamine agonist therapy” using KB220 variants in lowering methylation and increasing acetyl groups to develop DRD2 expression, even in DRD2 A1 allele carriers, subsequently leading to heightened DA function and decline in drug and non-drug seeking behaviors. This may even have relevance to Lyme carriers especially because of its effects on dopamine function. While we do not know the effects of Lyme on, for example, dopa-decarboxylase activity to produce dopamine, it seems important to at least experiment with a pro-dopamine regulator like KB220z in anxious and depressed patients.
